# Supplementary material for: Thrombophilia-Related Single Nucleotide Variants and Altered Coagulation Parameters in a Cohort of Mexican Women with Recurrent Pregnancy Loss
Source: Diagnostics (Basel). 2025 Dec 7;15(24):3111. doi: 10.3390/diagnostics15243111 (PMC12731847; doi:10.3390/diagnostics15243111)
Supplement: Supplementary file 1 [file diagnostics-15-03111-s001.zip › diagnostics-3986231-supplementary.pdf]

Supplementary Table S1. Descriptive statistics of the metabolites analyzed

Metabolite	Mean	Range	Standard deviation	Reference values
Folic acid (B9, ng/ml)	22.15	6.26-40	10.11	3.1-20.5
Cobalamin (B12, pg/ml)	599.38	107-3600	613.95	187-883
Homocysteine (µmol/L)	7.72	5-13.8	1.97	5-15
Fibrinogen (mg/dl)	339.27	242-627	70.92	183.1-454.9
Antithrombin III (%)	99.31	62-124	11.53	83-128
Thrombin time (sec)	15.94	12.2-19.8	1.55	13-15.8
Prothrombin time (sec)	12.77	9.5-23.4	1.65	9.7-13.3
Activated partial thromboplastin time (sec)	31.56	11.5-52.5	4.24	26.1-37.1
INR	1.03	0.82-1.23	0.068	0.8-1.2
D-dimer (ng/ml)	445.24	60-2110	355.98	<500
Factor XII activity (%)	76.8	44-103	15.45	52-175

Supplementary Table S2. Analysis of the Hardy-Weinberg equilibrium in the SNV studied.

SNV	Genotypes	Genotype Frequencies	Allele Frequencies	Fisher's exact test (p-value)
AGT rs4762	G/G	0.657	G: 0.819<br>A: 0.181	0.51
	G/A	0.324		
	A/A	0.019		
AGT rs699	G/G	0.705	G: 0.8335<br>A: 0.1665	0.48
	G/A	0.257		
	A/A	0.038		
F7 rs6046	G/G	0.762	G: 0.8715<br>A: 0.1285	0.67
	G/A	0.219		
	A/A	0.019		
FGB rs1800790	G/G	0.610	G: 0.791<br>A: 0.21	0.55
	G/A	0.362		
	A/A	0.029		
MTR rs1805087	A/A	0.238	A: 0.581<br>G: 0.419	<0.0001*
	A/G	0.686		
	G/G	0.076		
MTRR rs1801394	A/A	0.619	A: 0.776<br>G: 0.224	0.40
	A/G	0.314		
	G/G	0.067		
MTHFR rs1801133	G/G	0.200	G: 0.4715<br>A: 0.5285	0.44
	G/A	0.543		
	A/A	0.257		
MTHFR rs1801131	T/T	0.810	T: 0.891<br>G: 0.11	0.10
	T/G	0.162		
	G/G	0.029		
F2 rs1799963	G/G	0.99	G: 0.99<br>A: 0.01	0.0048*
	G/A	0		
	A/A	0.01		
F5 rs6025	C/C	0.981	C: 0.9905<br>T: 0.0095	1
	C/T	0.019		
	T/T	0		
F12 rs1801020	G/G	0.290	G: 0.558<br>A: 0.442	0.63
	G/A	0.536		
	A/A	0.174		
F13A1 rs5985	C/C	0.551	C: 0.7465<br>A: 0.2535	1
	C/A	0.391		
	A/A	0.058		
SERPINE1 rs1799889	5G/5G	0.010	5G: 0.367<br>4G: 0.633	<0.0001*
	5G/4G	0.714		
	4G/4G	0.276		

(*): These values imply a statistical significance at p<0.05, so it is inferred that these SNVs are not in equilibrium.

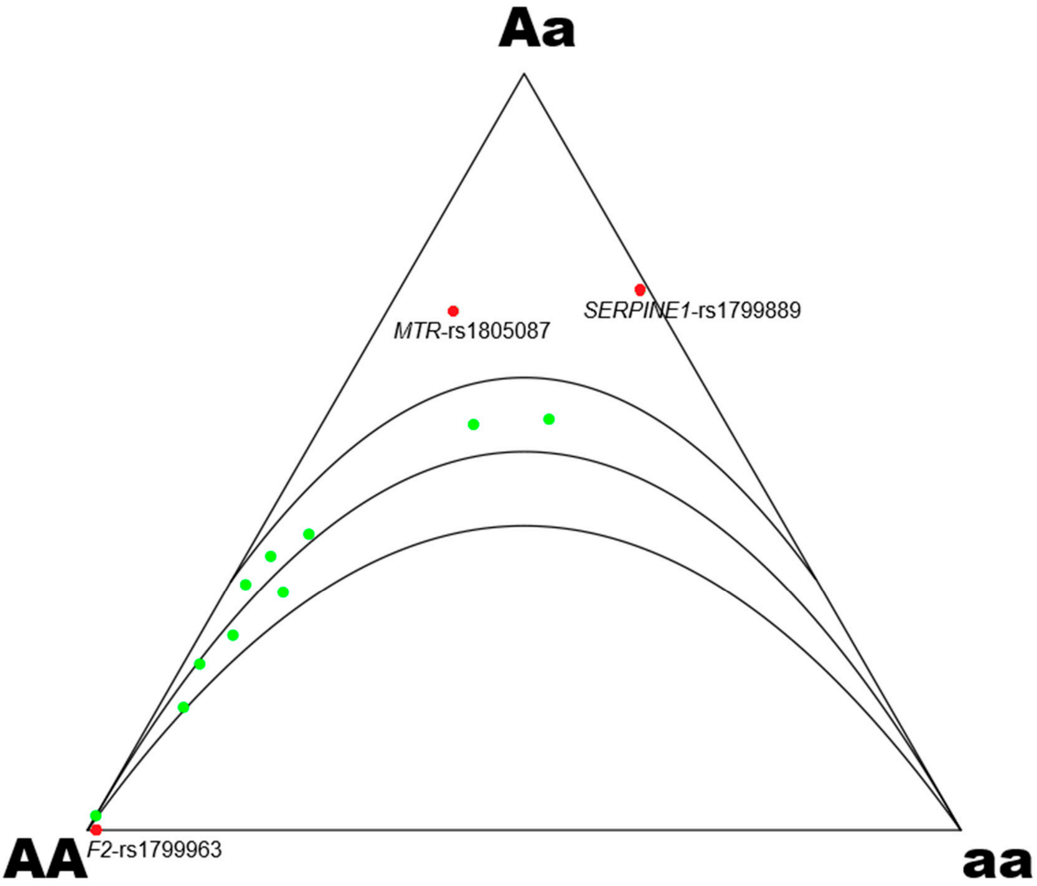

Supplementary Figure S1. Finetti diagram showing the distribution of genotype frequencies of the 13 SNVs analyzed. All SNVs in Hardy-Weinberg equilibrium are shown in green, while those that deviate from it are highlighted in red. (León, 2024; HardyWeinberg in R).

Supplementary Table S3. Inheritance models of the association of SNV *F7*-rs6046 with INR values.

Model	Genotype	n	Response mean (s.e.)	Difference (95% CI)	p-Value	AIC	BIC
Codominant	G/G	79	1.02 (0.01)	0.00	0.003	-262.9	-252.4
	G/A	21	1.06 (0.02)	0.04 (0.01-0.07)*			
	A/A	2	1.15 (0.02)	0.13 (0.03-0.22)*			
Dominant	G/G	79	1.02 (0.01)	0.00	0.0035	-261.6	-253.8
	G/A-A/A	23	1.07 (0.01)	0.05 (0.02-0.08)*			
Recessive	G/G-G/A	100	1.03 (0.01)	0.00	0.016	-258.9	-251
	A/A	2	1.15 (0.02)	0.12 (0.02-0.21)*			
Overdominant	G/G-A/A	81	1.02 (0.01)	0.00	0.031	-257.7	-249.8
	G/A	21	1.06 (0.02)	0.04 (0.00-0.07)*			
Additive	-	-	-	0.05 (0.02-0.07)*	0.0009**	-264.1	-256.2

s.e.: Standard error, CI: Confidence interval, AIC: Akaike information criteria, BIC: Bayesian information criteria.

Supplementary Table S4. Association of SNV *F12*-rs1801020 with the percentage of factor XII activity (%).

Model	Genotype	n	Response mean (s.e.)	Difference (95% CI)	p-Value	AIC	BIC
-	A/A	6	56.87 (5.53)	0.00	0.0001**	237.5	241.7
	G/A	24	81.79 (2.34)	24.92 (14.30-35.54)*			

s.e.: Standard error, CI: Confidence interval, AIC: Akaike information criteria, BIC: Bayesian information criteria.

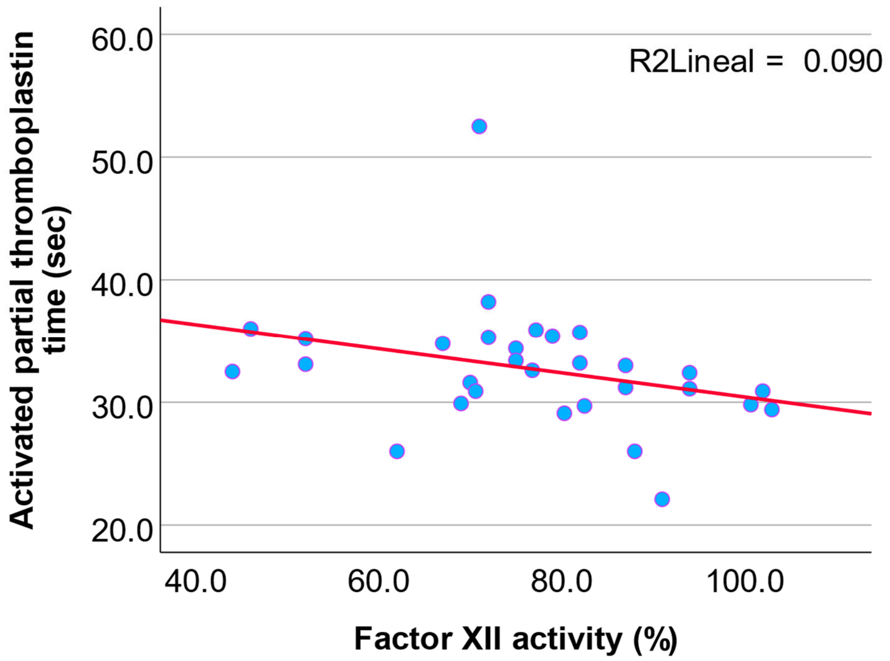

Supplementary Figure S2. Scatter plot showing the relationship between the percentage of factor XII activity and the activated partial thromboplastin time. The line whose slope best explains the dispersion of the data is highlighted in red. (León, 2024, IBM SPSS Statistics).
